# Cotard’s Syndrome in a Patient with Metastatic T-Cell Lymphoma: A Case of Walking Corpse Delusion

**DOI:** 10.1192/j.eurpsy.2026.11553

**Published:** 2026-07-31

**Authors:** F. Karsavurdan, O. Metin

**Affiliations:** Psychiatry, Akdeniz University Faculty of Medicine, Antalya, Türkiye

## Abstract

**Introduction:**

Nihilistic delusions (délires de négation) are rare beliefs that one is dead, non-existent, or decaying. Known as Cotard’s Syndrome (CS), or “walking corpse” syndrome, it has three forms: Type I, Type II, and psychotic depression subtype. Though clinically important, CS is poorly understood and rarely reported. Impaired connectivity in emotional and interoceptive brain areas may lead to alexisomia, alexithymia, and distorted bodily awareness. CS is associated with cerebral infarcts, frontotemporal atrophy, epilepsy, encephalitis, brain tumors, TBI, and nondominant hemisphere lesions.

**Objectives:**

To describe clinical features, possible pathophysiology, and treatment in a patient with metastatic T-cell lymphoma presenting with CS.

**Methods:**

Clinical case report and brief literature review

**Results:**

A 59-year-old man under inpatient care for metastatic angioimmunoblastic T-cell lymphoma developed nihilistic delusions. He had a history of head and neck squamous cell carcinoma treated with surgery and chemoradiotherapy. After four years’ remission, pancytopenia led to T-cell lymphoma diagnosis. Following CHOP, gemcitabine–oxaliplatin was started. By day 20 (CRP: 181), psychiatric symptoms appeared.

He was withdrawn, defensive, with reduced speech, psychomotor retardation, depressive affect, guilt delusions, severe anhedonia, amotivation, and poor oral intake. He stated: “I am already dead,” “I should have died three days ago,” “I am in the grave,” “If I had Alzheimer’s, I would die earlier,” and “Don’t waste time with me.” He lacked insight, had no hallucinations, was fully oriented, and attention was intact.

Labs showed pancytopenia and high liver enzymes. CT showed chronic ischemia; MRI revealed cerebral atrophy and infarcts, no mass lesion. MMSE: 20/30. No prior psychiatric or neurological illness.

The clinical picture matched psychotic depression subtype of CS. Sertraline (50 mg/day) and aripiprazole (2.5 mg/day) were started, but stopped on day 4 due to deterioration. He died three weeks later from malignancy-related arrhythmia.

**Conclusions:**

Cotard’s Syndrome is a rare neuropsychiatric disorder with distinct nihilistic delusions. It can emerge in systemic or neurological illness. Though multiple treatments exist, electroconvulsive therapy (ECT) remains the gold standard. Further studies are needed to better understand and manage CS.

**Disclosure of Interest:**

None Declared

